# Radiation-induced magnetization reversal causing a large flux loss in undulator permanent magnets

**DOI:** 10.1038/srep37937

**Published:** 2016-11-29

**Authors:** Teruhiko Bizen, Ryota Kinjo, Teruaki Hasegawa, Akihiro Kagamihata, Yuichiro Kida, Takamitsu Seike, Takahiro Watanabe, Toru Hara, Toshiro Itoga, Yoshihiro Asano, Takashi Tanaka

**Affiliations:** 1 JASRI, Koto 1-1-1, Sayo, Hyogo 679-5198, Japan; 2 RIKEN SPring-8 Center, Koto 1-1-1, Sayo, Hyogo 679-5148, Japan

## Abstract

We report an unexpectedly large flux loss observed in permanent magnets in one of the undulators operated in SACLA, the x-ray free electron laser facility in Japan. Characterizations of individual magnets extracted from the relevant undulator have revealed that the flux loss was caused by a homogeneous magnetization reversal extending over a wide area, but not by demagnetization of individual magnets damaged by radiation. We show that the estimated flux-loss rate is much higher than what is reported in previous papers, and its distribution is much more localized to the upstream side. Results of numerical and experimental studies carried out to validate the magnetization reversal and quantify the flux loss are presented, together with possible countermeasures against rapid degradation of the undulator performance.

In synchrotron radiation (SR) and x-ray free electron laser (XFEL) facilities, magnetic devices to periodically deflect high-energy electrons are installed, which are composed of south- and north-pole magnets arranged alternately, and are usually referred to as undulators. Because its periodic length is typically of the order of several centimeters or shorter, permanent magnets (PMs) with high remanence are usually chosen instead of electromagnets to obtain a strong magnetic field enough to generate bright SR and achieve lasing in XFELs.

Because the undulators are operated in a high-energy accelerator, the PMs are continuously exposed to high-dose radiation such as high-energy electrons, synchrotron x rays, neutrons and *γ* rays. As a result, they can be more or less damaged and some amount of magnetic flux may be lost, which varies the magnetic field along the electron beam path and deteriorates the undulator performance. Because the flux loss generally progresses with the increased radiation dose, it is important to predict the lifetime of an undulator, i.e., the period of how long it can be operated with the performance being kept within required specifications. It should be noted, however, that the radiation-induced flux loss is a complicated process; it depends on a huge number of parameters related to the PM specifications as well as the electron beam parameters, and thus a theoretical prediction of the flux-loss rate is almost impossible. This is the reason why many efforts have been devoted to studying the radiation damage of PMs experimentally.

The above studies can be divided into two types. One is to measure the flux-loss rate of undulators actually in operation, as a function of absorbed dose measured by radiation monitors such as thermo-luminescent dosimeters^1^,^2^,^3^,^4^,^5^. The other is to measure the flux-loss rate of sample PMs irradiated with a high-energy (>2 GeV) electron beam as a function of the number of incident electrons^6^,^7^,^8^,^9^. The former studies have revealed that the absorbed dose of 10^4^~10^5^ Gy leads to a flux loss of 1%, while the latter ones have demonstrated that exposure to 10^14^~10^15^ high-energy electrons causes the same amount of flux loss. Although it is not straightforward to convert the number of incident electrons to the absorbed dose, we have confirmed through numerical studies that the above two experimental results to quantify the flux-loss rate are consistent; another numerical study^3^ has also found that loss of 6.25 × 10^14^ electrons with the energy of 7 GeV leads to the absorbed dose of 10^4^~10^6^ Gy near the beam axis, which is roughly consistent with the above discussion.

It is worth noting that the flux loss of PMs caused by radiation is usually resulted from demagnetization, which can be divided into two types: reversible and irreversible ones. In the former, the directions of the magnetic domains pointing to the same direction before irradiation, become different and random, resulting in cancellation of magnetic fields generated by individual domains. In the latter, the crystalline structure having magnetic anisotropy is modified or destroyed, leading to an irreversible damage of PMs. It is well known that the demagnetization observed in an electron accelerator usually comes from the former mechanism.

In SACLA^10^, an XFEL facility being operated since 2011 in Japan, a beam halo monitor has been developed^11^,^12^ to routinely monitor the number of halo electrons incident on the undulator PMs, and installed in front of the undulator beamline. It is a diamond-based detector operated in an ionization mode, and has a lower detection limit of 2 × 10^3^ electrons/pulse or better. Together with the experimentally determined flux-loss rate mentioned above, it is in principle possible to precisely predict the undulator lifetime. It should be noted, however, that we need to know the distribution of the flux loss along the longitudinal axis in addition to its maximum value to evaluate the performance degradation. Useful information is found in a number of refs 3, 4, 5 reporting that the flux loss has been observed almost over the whole undulator length, giving rise to a long-range variation of the peak field.

With the above discussions regarding the rate and distribution of the radiation-induced flux loss, the undulator lifetime can be predicted reasonably well. As an example, we define the tolerance of the maximum flux loss to be 1%. This results in a maximum phaser error of 180 degree in the SACLA undulators, if the peak field linearly changes from the entrance to the exit because of the radiation-induced flux loss. The charge of the halo electrons incident on the SACLA undulators measured by the halo monitor (*q*_*he*_) has been found to be less than 1 fC per pulse in the normal operation, i.e., without any elements to intercept the electron beam such as profile monitors. This amounts to 4 × 10^−6^ of the typical bunch charge of 250 pC in SACLA, meaning that the halo electrons and dark currents are well suppressed by a magnetic chicane located just in front of the undulator. Recalling the nominal repetition rate of 60 Hz and assuming that the accelerator is operated for 7000 hours per year, the undulator lifetime has been expected to be roughly around 10 years even in the worst case.

In this paper, we report a large flux loss observed recently in the SACLA undulators, which is controversially large when compared to the above discussions generally accepted. We show that the flux loss is caused by a homogeneous magnetization reversal (MR) extending over a wide area, in which all magnetic domains are reversed with respect to the original state. This is in contrast to the usual demagnetization explained above. A simple model is proposed to explain the mechanism of how it happens, together with an effective and practical method to impede the progress of the flux loss and thus improve the undulator lifetime.

## Results

### Flux-loss measurements in SACLA undulators

SACLA started operation in 2011 with a hard x-ray FEL beamline accommodating 18 undulator segments to provide intense and short x-ray laser pulses. All undulators are composed of Neodymium-Iron-Boron (NdFeB) rare earth magnets with the intrinsic coercivity (Hcj) of 2 MA/m and remanence of 1.25 T, and have the same specifications of the magnetic period, length and maximum flux density of 18 mm, 5 m and around 1.3 T, respectively. Before installation in the accelerator tunnel of SACLA, the performance of each undulator was examined by measuring the magnetic field distribution along the longitudinal axis. Then all the segments have been arranged in line with a spacing of 1.15 m in between.

**Figure 1:**
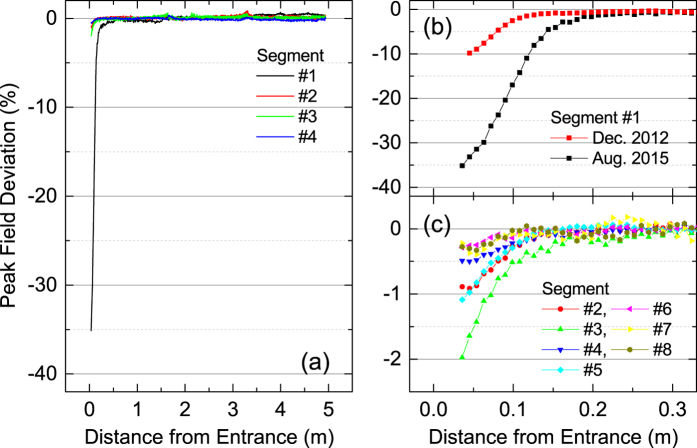
Results of the flux-loss measurement in the SACLA undulators. Each figure shows the peak-field variation at each magnet pole as a function of the distance from the entrance. Measured data over the whole undulator length are shown in (**a**), while details near the entrance are shown in (**b**) and (**c**).

In order to examine the flux loss of SACLA undulators after 4 years since it started operation, we measured the field distribution along the longitudinal axis during the maintenance period in August 2015. This can be done on site without moving the undulators to a dedicated facility, by means of a portable and precise magnetic measurement system^13^. We carried out the measurements at the minimum gap of 3.5 mm for eight undulator segments from the most upstream side. The results are shown in Fig. 1(a)~(c), where the magnetic flux loss, defined as the variation of the peak magnetic field at each magnet pole, is plotted as a function of the distance measured from each undulator entrance. Figure 1(a) shows the measurement results over the whole undulator length for the first four segments, while details near the entrance are shown as black squares in Fig. 1(b) for the 1st segment, and in Fig. 1(c) for the other (from 2nd to 8th) segments, respectively. Note that the ordinate scale is changed to facilitate visualization in Fig. 1(c).

The flux loss observed in the 1st segment is the most significant; it exceeds 30% near the entrance and decays rapidly as the distance from the entrance, suggesting that PMs themselves work to protect other PMs located downstream. This trend also applies to other segments, although the maximum flux loss is much lower. It should be emphasized that the rapidly decaying flux loss observed in the SACLA undulators is in contrast to the long-range flux loss reported in the former references as mentioned in Introduction.

Let us discuss when the large flux loss happened, especially in the 1st segment. In the initial commissioning period of XFELs before achieving a steady-state laser operation, many accelerator parameters are not optimized and thus the probability for the halo electrons and mis-steered core electrons to hit the undulator PMs is much higher. This means that the initial commissioning operation can potentially cause a large flux loss. In SACLA, such a commissioning operation continued by the end of 2011, when the laser saturation has been finally achieved. In December 2012, after one year since then, we measured the flux loss for the first time after the initial commissioning, the result of which is shown as red squares in Fig. 1(b). Comparing the two measurement results, we can conclude that the flux loss progressed continuously, and it cannot be accounted for by the initial commissioning operation.

**Figure 2:**
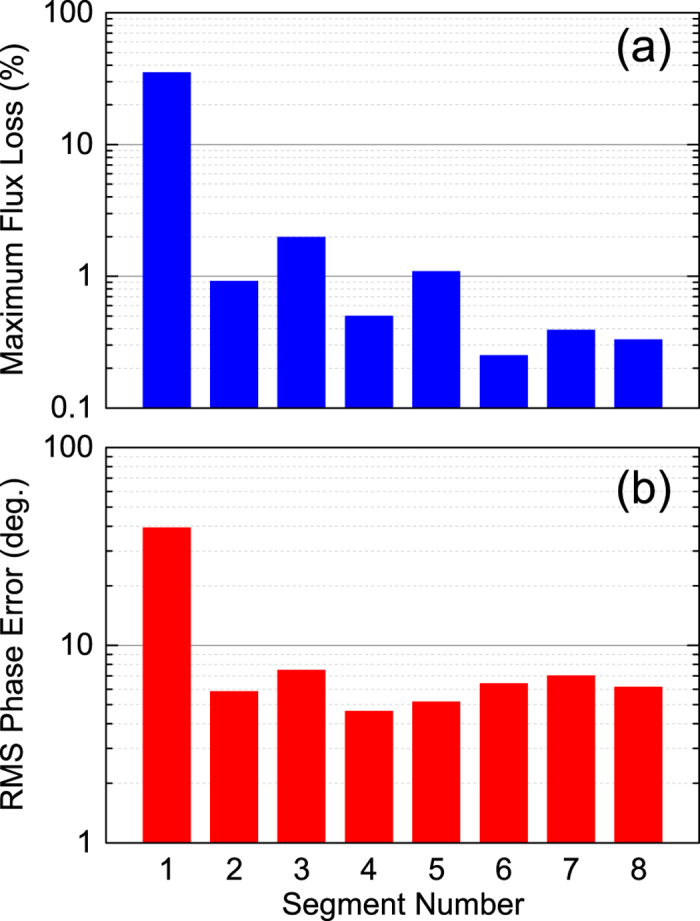
Summary of the flux-loss measurement; (**a**) the maximum flux loss and (**b**) evaluated phase error are plotted as a function of the segment number.

Figure 2(a) summarizes the maximum flux loss observed for each segment, showing that a larger segment number generally results in a lower flux loss. This suggests that an undulator itself works to protect other undulators downstream, like the radiation protection by individual PMs. It should be noted, however, that the reduction of the flux loss is not a simple function of the segment number; it exhibits an oscillatory behavior, which may be related to the betatron oscillation of the electron beam.

The induced flux loss described above may cause undulator performance degradation, which can be defined by the phase error evaluated from the magnetic field distribution measured along the longitudinal axis^14^. The evaluated phase errors for the eight segments are shown in Fig. 2(b). Note that the phase errors of SACLA undulators before installation have been confirmed to be around 5 degree in average. Comparing with Fig. 2(b), we can conclude that the effects due to the flux loss on the phase error are negligible except for the 1st segments that encountered a large flux loss, and the resultant phase error exceeds 30 degree.

Let us now discuss the validity of the large flux loss above 30% observed near the entrance of the 1st undulator segment. For this purpose, we need to know the total charge of halo electrons (*Q*_*he*_) actually incident on the PM. This can be done by integrating *q*_*he*_ measured by the halo monitor for each electron bunch over the whole period of operation. It should be noted, however, that there are several periods of time when the halo monitor has not been ready for measurement. We thus estimate as *Q*_*he*_ = *Q*_*t*_ × *q*_*he*_/*q*_*t*_, where *Q*_*t*_ is the total electron charge generated and accelerated in SACLA since March 2011 when it started operation until July 2015, while *q*_*he*_ and *q*_*t*_ are the halo and total electron charges during the period when the halo monitor is available. To be specific, *Q*_*t*_ and *q*_*t*_ measured by a general current transformer are 0.38 C and 0.18 C, and *q*_*he*_ measured by the halo monitor is 0.7 *μ*C, and thus *Q*_*he*_ is estimated to be 1.5 *μ*C, corresponding to 9 × 10^12^ halo electrons incident on the PM. This results in an expected flux loss of 0.1%, assuming the typical flux-loss rate mentioned in Introduction. Thus the flux loss over 30% observed in the 1st undulator segment in SACLA is a few orders of magnitude higher than what is predicted from the known experimental results.

### Characterization of individual PMs

**Figure 3:**
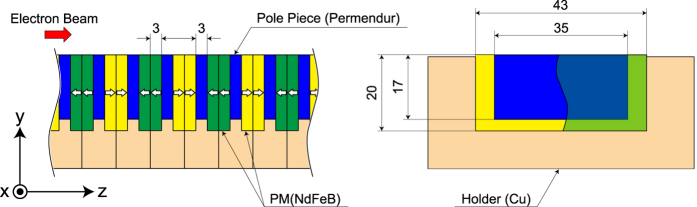
Schematic drawing of the magnetic array of the SACLA undulator. Dimensions are in mm.

To explore the reason for the large discrepancy between the measured and predicted flux losses, we extracted several PMs from the magnetic array near the entrance of the 1st undulator segment and carried out a detailed characterization by measuring the flux density ***B*** near the surface of each PM. Figure 3 shows a schematic drawing of the bottom half of the magnetic array of the SACLA undulator, which is composed of longitudinally magnetized PMs and pole pieces made of Permendur, a cobalt-iron soft magnetic alloy having the saturated flux density of 2.35 T. The empty arrow indicates the direction of magnetization of each PM and three coordinate axes are introduced for the following discussions. Note that the halo electrons are not directly incident on the PMs; there are several auxiliary components mainly made of copper near the entrance and exit of the undulator. The total length of them is nearly 40 mm, which is 2.8 times the radiation length and is long enough for the halo electrons to produce electromagnetic showers.

**Figure 4:**
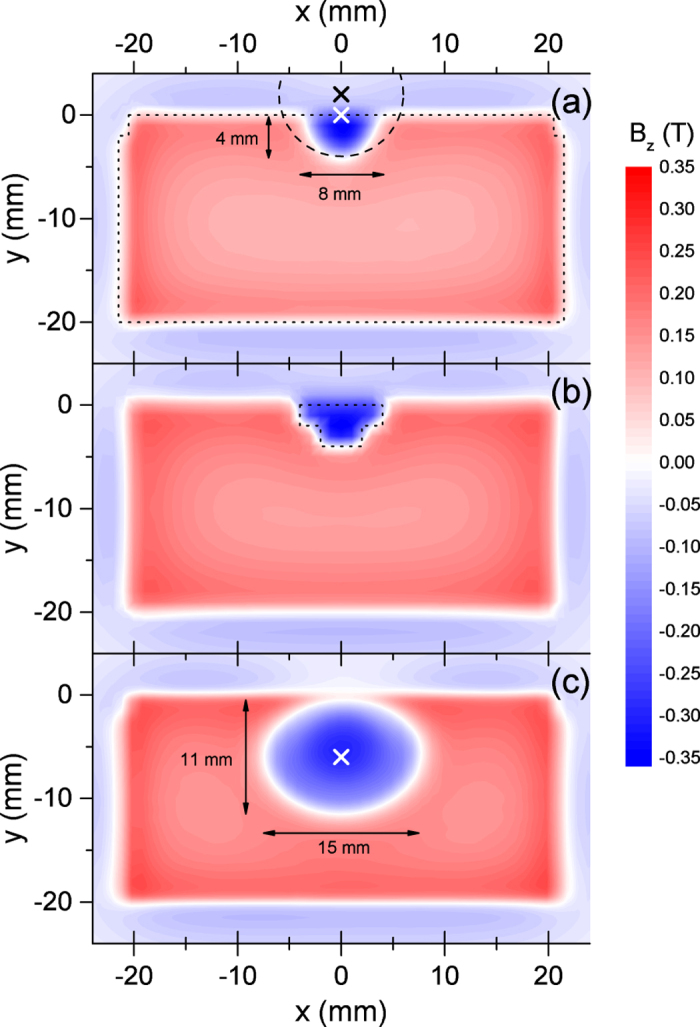
Contour plots of *B*_*z*_ near the surface of the PM; (**a**) measured for the PM extracted from the entrance of the SACLA undulator (1st segment), (**b**) computed with the assumption that the area surrounded by the dashed line is initialized by the electron bombardment, and (**c**) measured for the PM irradiated with the electron beam in the synchrotron.

The measurement has been done by sweeping a Hall-effect magnetic sensor over each PM at the position of *z* = 0.74 mm, where the origin of *z* axis is defined as the surface of the PM. The result is shown in Fig. 4 as a contour plot of the longitudinal component of ***B** (B*_*z*_) measured over the region wider than the cross-sectional area of the PM, whose visible outline is indicated by the dotted line. The origin of *y* axis is defined as the top edge of the PM, and the electron beam position in the normal operation is indicated by a symbol (black ×) for reference, which is nearly 2 mm over the top edge of the PM (*y* = 2 mm). Note that the RMS electron beam size is less than 0.03 mm in SACLA, which is much smaller than this reference symbol.

We find a semicircle region with the radius of 4 mm and area of 25 mm^2^, where the sign of *B*_*z*_ is reversed with respect to those in the other region, suggesting that the magnetization vector of the PM is reversed there. In other words, all the magnetic domains contained in this region are homogeneously reversed. It is obvious that such MR extending over a wide area, as opposed to the simple demagnetization of PMs, gives rise to a larger flux loss as observed in the SACLA undulator. In fact, we verified through numerical computations using a magnetostatic computation code RADIA^15^ that completely reversing the magnetization in the rectangular region with the aspect ratio of 2:1 (*x:y*) and area of 25 mm^2^ results in the flux loss of 33%, which is in good agreement with what has been actually measured. Note that the rectangular shape has been chosen instead of a semicircle to facilitate the numerical process, which obviously has negligible impacts on the computation results.

It should be the emphasized that the center of the MR semicircle (indicated by white ×) is roughly located at the top edge of the PM (*y* = 0), but does not coincide with the center of the electron beam (*y* = 2 mm). This is obvious by drawing a circle with its center at the electron-beam center, as indicated by the dashed line in Fig. 4(a), and is easily understood by recalling that the halo electrons are first incident on the copper components located in front of the PMs; most of them will hit the top edge and produce electromagnetic showers whose distribution is axisymmetric roughly around (*x, y*) = (0, 0). This eventually means that half of the electromagnetic showers do not contribute to MR at least near the undulator entrance.

### Numerical validation

Because the MR observed in the SACLA undulator has never been reported elsewhere to our knowledge, it is scientifically important to discuss the mechanism of how it happens. It is well known that the PMs in the undulator are usually subject to strong reverse magnetic fields generated by other PMs, in addition to the self-demagnetizing field. As shown in Fig. 3, a group of PMs painted green are subject to reverse magnetic fields generated by another group painted yellow, and vice versa. Furthermore, the pole pieces sandwiched by the PMs can work to locally enhance the reverse field for the both groups. The strong reverse field, however, has negligible impacts on the undulator performance under a normal condition, because the coercivity of PM material chosen for the SACLA undulator is sufficiently high.

As discussed in previous studies^16^,^17^,^18^,^19^, bombardment of a high-energy electron induces thermal spikes along its path, which leads to localized temperature rise in a specific region in the PM. If the resultant temperature goes beyond the Curie point, the permanent magnetism is completely lost around this region. Even in the outer region where the temperature rise is not that high, the reduced coercivity will cause a magnetic domain reversal. As a result, it is reasonable to consider that the bombardment of high-energy electrons works to “initialize” the magnetization of the relevant region in the PM. It is obvious that this initialization is a transient process and the relevant region will be eventually relaxed to an equilibrium state, in which the magnetization condition will be determined by the magnetic field to which the region is subject.

Based on the above discussion, we carried out numerical studies using a simple model that a certain region of a PM is initialized by the electron bombardment. In other words, the PM material in this region reduces to a soft magnetic material having the saturated magnetic flux density identical to the remanence of the PM. Figure 4(b) shows the spatial distribution of *B*_*z*_ computed with RADIA under this assumption, with the dotted line indicating the border of the initialized region. Note that a convex polygon having the same area has been chosen instead of a semicircle to facilitate the numerical process, which obviously has negligible impacts on the result of computation. We find that the agreement between the measured and computed results is fairly well, suggesting the validity of the above discussion.

### Experimental quantification

**Figure 5:**
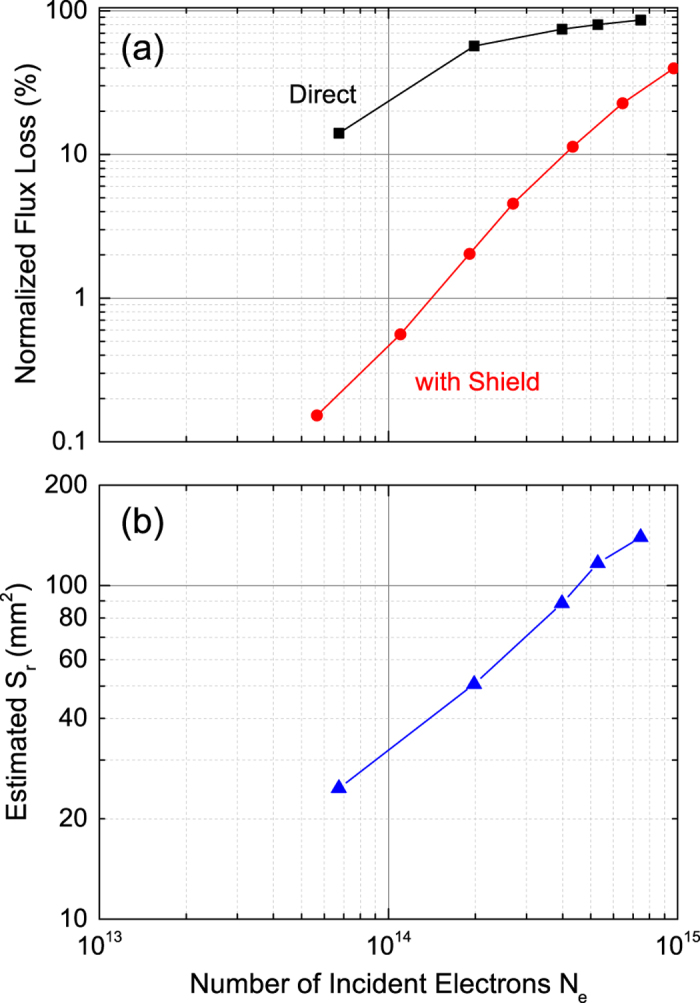
Results of irradiation experiments. The observed flux loss is normalized and plotted as a function of the number of incident electrons *N*_*e*_.

Apart from the mechanism of how the MR occurs, it is important to experimentally quantify the flux-loss rate of undulator PMs exposed to electrons to predict the undulator lifetime. For this purpose, we irradiated a test sample with an 8-GeV electron beam accelerated in a synchrotron located near SACLA, and measured the induced flux loss. The sample is composed of two elements; one is a 40-mm long copper block to model the components placed in front of the undulator PMs, while the other is a 1.5-period undulator with the specifications same as those of the SACLA undulator, except that only the bottom half is used for the experiments. The position of the center of the electron beam injected to the sample is 6 mm below the top edge of the sample. This is to make sure that all electrons are incident on the sample. After being exposed to the 8-GeV electron beam for several hours, the magnetic field distribution along the *z* axis was measured by sweeping a Hall-effect sensor 4 mm above the top edge of the sample. The flux loss is defined as the variation of the peak field generated by the central magnet pole. This process has been repeated several times to measure the flux loss as a function of the number of incident electrons. The results are shown as black squares in Fig. 5(a), where the flux losses are normalized by the peak field measured before starting irradiation.

After exposed to 7.4 × 10^14^ electrons, one of the two PMs consisting the central pole was extracted, and *B*_*z*_ near the surface was measured in the same manner as described before. The result is shown in Fig. 4(c), in which MR was observed in an elliptical region with the major and minor axes of 15 mm and 11 mm, whose area is roughly five times larger than that observed in the SACLA undulator.

Let us compare the two experimental results observed in the SACLA undulator and in the magnet sample irradiated in the synchrotron. To facilitate the following discussions, the former experimental condition is referred to as the condition (i), while the latter is referred to as the condition (ii). Note that the typical gap between the top and bottom magnetic arrays has been 4 mm in the former, while only the bottom magnetic array was irradiated in the latter. For a quantitative comparison, we need to take into account the difference in the irradiation condition between the two. To be specific, the PM in the former is exposed to halo electrons of an electron beam with its core passing between the top and bottom magnetic arrays, while that in the latter is exposed to core electrons of an electron beam with its center located 6 mm below the top edge. This leads to the difference in the position and shape of the MR region as shown in Fig. 4(a,c), which can cause some difference in the flux loss measured above the top edge of the PM. We thus introduce another index to specify the effects due to irradiation, i.e., the area of the MR region *S*_*r*_, which is explained as follows.

It is reasonable to assume that the MR region expands with the increased number of incident electrons (*N*_*e*_), with its position and aspect ratio being kept constant. Under this assumption, *S*_*r*_ to cause a certain flux loss can be numerically estimated; for example, the flux losses measured in the condition (ii) shown in Fig. 5(a) (black squares) are converted to *S*_*r*_ and plotted as a function of *N*_*e*_ in Fig. 5(b). Roughly speaking, *S*_*r*_ linearly increases as *N*_*e*_ in this condition.

Using *S*_*r*_ estimated from the experimental data, the two conditions (i) and (ii) can be compared in terms of the rate *ρ*_*r*_ = *S*_*r*_/*N*_*e*_ at which the MR region expands. Substituting the experimental data described so far, we have *ρ*_*r*_ = 50 *mm*^2^/10^13^*e*^−^ in the condition (i), and *ρ*_*r*_ = 3 *mm*^2^/10^13^*e*^−^ in the condition (ii). Note that we have taken into account that half of the electromagnetic showers produced in the condition (i) do not contribute to MR as explained before, and we have made an assumption that *S*_*r*_ linearly increases as *N*_*e*_ in the both conditions (i) and (ii). Although there are several experimental factors that may cause numerical errors in the experimental data, the above discrepancy is too large to be explained by these errors. It is thus reasonable to say that the MR region in the condition (i) expanded much faster than that in the condition (ii).

Before discussing the reason for the above discrepancy, we describe a method to improve the SACLA undulator lifetime, which is extremely important from a practical point of view. One simple idea is to put a radiation shield in front of the PMs. Note that its effectiveness is validated by the fact that the flux loss in the SACLA undulators occurred in a narrow range near the entrance as shown in Fig. 1(b). In order to experimentally demonstrate the effects due to a radiation shield, we measured the flux loss of the test sample irradiated in the same manner as in the condition (ii), but shielded by a 100-mm thick block made of stainless steel. The results are shown as red circles in Fig. 5(a), where we find the radiation shield significantly impedes the progress of the flux loss. For example, the flux loss induced by ~5 × 10^13^ incident electrons is as low as 0.1%, nearly two orders of magnitude less than that without the shield. It should be emphasized, however, that the simple radiation shield described above is not necessarily effective in other cases when the flux loss is not localized near the entrance and extends over a wide range.

Another method to reduce the flux loss and improve the undulator lifetime, which should be mentioned besides the radiation shield described above, is to make use of PMs with higher radiation hardness, i.e., Samarium-Cobalt (Sm_2_Co_17_) magnets instead of NdFeB magnets. In order to compare the two PM materials in terms of the flux loss, we repeated the irradiation experiments using SmCo magnets with the remanence of 1.1 T and intrinsic coercivity of 2.2 MA/m in the condition (ii). Even with *N*_*e*_ exceeding 10^15^, which caused a large flux loss of nearly 100% in NdFeB magnets, the flux loss of SmCo magnets has been found to be negligibly small. This experimental results remind us of the great advantage of SmCo magnets in terms of the radiation hardness, although NdFeB magnets are superior in many other points such as the remanence, easiness of handling, and manfucaturing cost.

## Discussion

Let us now discuss the reason why the MR region expanded much faster in the condition (i) than in (ii). Among many possible factors affecting the growth rate of *S*_*r*_, we focus on the strength of the reverse field and its distribution over the PM.

It is well known that radiation-induced demagnetization of a PM strongly depends on its shape, or more specifically, the self-demagnetizing field strength^20^. This means that the magnetic domain can be more easily reversed by irradiation when it is subject to a higher reverse field. For example, former experimental studies^6^ using two different PMs, whose typical self-demagnetizing field strengths are 0.8 T and 0.4 T, have revealed that the former PM has demagnetized more than the latter by a factor of nearly 5, suggesting that the reverse field plays a key role in the process of demagnetization and thus MR.

**Figure 6:**
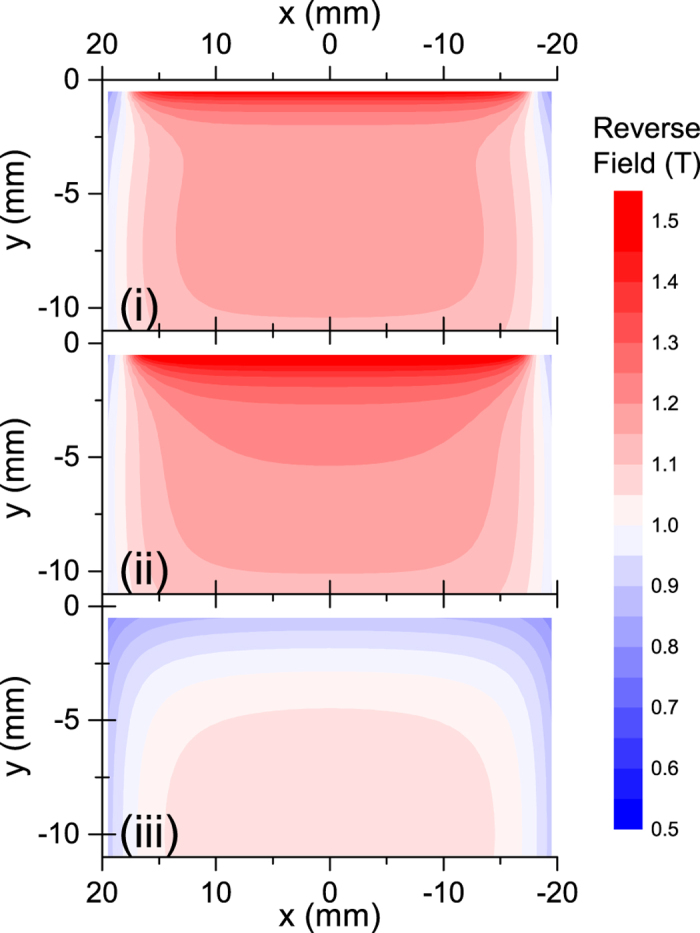
Spatial distribution of the the reverse field in the three conditions (i)~(iii).

It should be also noted that PMs in the undulator magnetic array are subject to a reverse field higher than the self-demagnetizing field, which is locally enhanced by the pole pieces. For example, Fig. 6 shows the reverse field computed in the *x*-*y* plane for the conditions (i) and (ii), together with the simplest case when only a single piece of PM having the same specifications is assumed, which is referred to as the condition (iii). It is found that the reverse fields in the conditions (i) and (ii) are higher than those in (iii) over the whole region, and is locally enhanced near the top edge.

Note that the pole pieces made of Permendur as shown in Fig. 3 are substituted by “virtual PMs” with the same dimensions, in computing the reverse field in Fig. 6; they are magnetized along the *y* axis with the remanence corresponding to the saturated flux density of Permendur, and are assumed to be free from MR. Such a simplification using virtual PMs makes it possible to compute the magnetic field semi-analytically, and significantly reduces the numerical cost. Although the absolute value computed under this simplification may be somewhat different from the real value, the relative accuracy of the reverse field is supposed to be reliable enough for qualitative comparisons between the conditions (i)~(iii).

**Figure 7:**
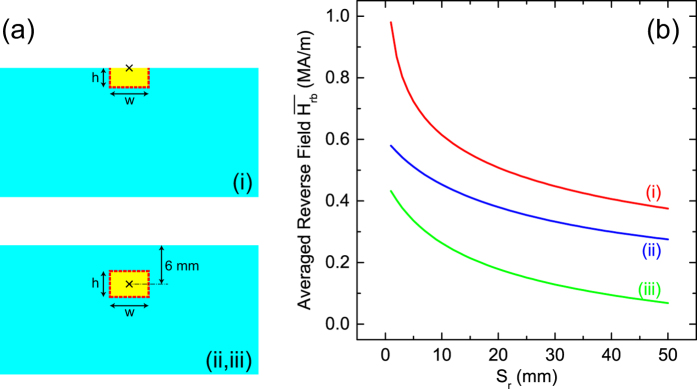
Consideration of the reverse field in the three conditions (i)~(iii): (**a**) simplified configurations of the MR regions, and (**b**) the averaged reverse field 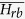 plotted as a function of *S*_*r*_.

To compare the reverse-field distributions in terms of the MR region, we consider two configurations for the MR experimentally observed, which are indicated by yellow rectangles shown in Fig. 7(a). Note that both regions are simplified to rectangles having the aspect ratios of *h/w* = 2 in (i) and *h/w* = 1.36 in (ii). The symbol × indicates the central position of the electromagnetic shower, and thus the onset point of MR for each condition, around which the MR region expands.

Because the MR region expands when the magnetic domains near its border are reversed, the expansion rate *ρ*_*r*_ is expected to be strongly correlated with the reverse field along the border (*H*_*rb*_). We thus compute its average 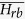 along the border of the MR region indicated by red dashed lines in Fig. 7(a), as an index to consider the expansion process of the MR region.

Figure 7(b) shows 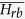 computed as a function of *S*_*r*_ in the three conditions (i)~(iii). Note that the MR region in the condition (iii) is assumed to expand in the same manner as in the condition (ii). In the whole range of interest, we have 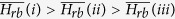, where 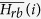 denotes 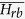 computed in the condition (i), etc. This relation, which is universal under practical conditions, can be easily understood by recalling the reverse-field distribution shown in Fig. 6.

Let us assume that the relation between *ρ*_*r*_ and 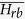 can be given by a simple formula, 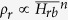, where *n* can be roughly estimated using the experimental and numerical data. Recalling that *ρ*_*r*_ roughly satisfies the relation 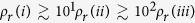, it is reasonable to say that *n* may be around 3~4. In other words, the expansion of the MR region is a highly nonlinear process with respect to the reverse field in the PM. Such a nonlinearity gives rise to an unexpectedly larger flux loss which cannot be predicted by simply extrapolating the conventional experimental data, as has been observed in the SACLA undulator.

Because the reverse field is in general higher for thinner PMs, this issue may be much more critical for shorter undulator periods. It is thus important to design an undulator magnetic circuit to reduce the reverse field as much as possible, especially at the positions near the electron beam, which has not been seriously considered in conventional undulators with the period longer than, e.g., 20 mm.

## Additional Information

**How to cite this article**: Bizen, T. *et al*. Radiation-induced magnetization reversal causing a large flux loss in undulator permanent magnets. *Sci. Rep.* **6**, 37937; doi: 10.1038/srep37937 (2016).

**Publisher's note:** Springer Nature remains neutral with regard to jurisdictional claims in published maps and institutional affiliations.
